# Multimodal mental health analysis in social media

**DOI:** 10.1371/journal.pone.0226248

**Published:** 2020-04-10

**Authors:** Amir Hossein Yazdavar, Mohammad Saeid Mahdavinejad, Goonmeet Bajaj, William Romine, Amit Sheth, Amir Hassan Monadjemi, Krishnaprasad Thirunarayan, John M. Meddar, Annie Myers, Jyotishman Pathak, Pascal Hitzler

**Affiliations:** 1 Department of Computer Science, Kansas State University, KS, United States of America; 2 Department of Computer Science & Engineering, Ohio State University, OH, United States of America; 3 College of Engineering and Computing, University of South Carolina, SC, United States of America; 4 Department of Artificial Intelligence & Computer Engineering, University of Isfahan, Isfahan, Iran; 5 Department of Biological Sciences, Wright State University, OH, United States of America; 6 Department of Computer Science and Engineering, Wright State University, OH, United States of America; 7 Department of Health Care Policy and Research, Weill Cornell Medicine, Cornell University, New York, NY, United States of America; Beihang University, CHINA

## Abstract

Depression is a major public health concern in the U.S. and globally. While successful early identification and treatment can lead to many positive health and behavioral outcomes, depression, remains undiagnosed, untreated or undertreated due to several reasons, including denial of the illness as well as cultural and social stigma. With the ubiquity of social media platforms, millions of people are now sharing their online persona by expressing their thoughts, moods, emotions, and even their daily struggles with mental health on social media. Unlike traditional observational cohort studies conducted through questionnaires and self-reported surveys, we explore the reliable detection of depressive symptoms from tweets obtained, unobtrusively. Particularly, we examine and exploit multimodal big (social) data to discern depressive behaviors using a wide variety of features including individual-level demographics. By developing a multimodal framework and employing statistical techniques to fuse heterogeneous sets of features obtained through the processing of visual, textual, and user interaction data, we significantly enhance the current state-of-the-art approaches for identifying depressed individuals on Twitter (improving the average F1-Score by 5 percent) as well as facilitate demographic inferences from social media. Besides providing insights into the relationship between demographics and mental health, our research assists in the design of a new breed of demographic-aware health interventions.

## Introduction

Depression is a highly prevalent public health concern and a major cause of disability worldwide. Depression affects 6.7% (i.e., about 16 million) Americans each year [1]. According to the World Mental Health Survey conducted in 17 countries, about 5% of people reported having at least one depressive episode in 2011 [2]. Untreated or undertreated depressive symptoms can lead to suicide and other chronic and risky behaviors such as drug or alcohol addiction [3]. More than 90% of people who commit suicide have a pre-existing diagnosis of depression [4].

Global efforts to curb depression involve identifying depressive symptoms through survey-based methods employing online questionnaires. These approaches suffer from under-representation as well as sampling bias. Survey data also exhibit problems due to temporal gaps between the data collection and dissemination of findings.

Recent years have witnessed rapid growth in the analysis of social media for studying a wide range of health problems from detecting the influenza epidemic [5] and cardiac arrest [6] to studying mood and mental health conditions [7, 8]. The widespread adoption of social media where people voluntarily and publicly express their thoughts, moods, emotions, and feelings, and share their daily struggles with mental health has not been adequately tapped into studying mental illnesses, such as depression. Insights gleaned from social media such as Twitter can be complementary to the current survey-based methods that can assist both governmental and non-governmental organizations in policy development.

The visual and textual content shared on different social media platforms like Twitter offer new opportunities for a deeper understanding of self-expressed depression both at an individual and community-level. For instance, the news headline “Twitter Fail: Teen Sent 144 Tweets Before Committing Suicide & No One Helped” highlights the need for better tools for gleaning useful insights from user generated content on social media platforms that can assist policy designers in providing resources for individuals with depressive symptoms. Recent analyses have lead to data-driven discoveries alongside the traditional hypothesis-testing social science process [9]. They have suggested that language style, sentiment, users’ activities, and engagement expressed in social media posts can predict the likelihood of depression [10, 11]. These studies often use psycholinguistic analysis, supervised and unsupervised language modeling, and expressed topics of interest. However, except for a few attempts, [12–15], these investigations have seldom studied extraction of emotional state from the visual content of posted images and profile images. Visual content can express users’ emotions more vividly, and psychologists have noted that imagery is an effective medium for communicating difficult emotions.

According to eMarketer [16], photos accounted for 75% of content posted on Facebook worldwide, and are the most engaging type of content (87%). Indeed, “a picture is worth a thousand words” and now, “photos are worth a million likes.” Similarly, on Twitter, the tweets with image links get twice as much attention as those without [17], and video-linked tweets drive up engagement [18]. The ease and naturalness of expression through visual imagery can serve to glean depressive symptoms in vulnerable individuals who often seek social support through social media [19]. Further, as psychologist Carl Rogers highlights, we often pursue and promote our Ideal-Self. In this regard, the choice of profile image can be a proxy for one’s online persona [20], providing a window into an individual’s mental health status. For instance, choosing a profile image with the emaciated legs of an individual with several cuts portrays negative self-view [21]. Moreover, psychologists have argued that people use pictures to communicate messages in social media posts which represent our “Ideal Self”, or who we want to be. Indeed, we are constantly motivated to pursue behaviors that bring us closer to our Ideal Self.

Inferring demographic information like gender and age can be crucial for stratifying our understanding of population-level epidemiology of mental health disorders. Relying on electronic health records data, previous studies have explored gender differences in depressive behavior from different angles including prevalence, age of onset, comorbidities, as well as biological and psychosocial factors. For instance, women have been diagnosed with depression twice as often as men, [22] and a national psychiatric morbidity survey in the UK has shown a higher risk of depression in women [23]. On the other hand, suicide rates for men are three to five times higher compared to women [24]. Women are more likely to socialize and express their dysphoria, while men tend to express their anger and show negative behaviors such as alcohol abuse and drug dependency [25].

Although depression can affect anyone at any age, the signs and risk factors for depression vary for different age groups [26]. Depression triggers for children include domestic violence, and loss of a pet, or family member. For adolescents, depression may arise from hormonal imbalances [27].

Late-life depression has caused the suicide rate in people aged 80 to 84 to be more than twice that of the general population [28]. Depression in the elderly population often occurs with other medical conditions that persist, which can increase the risk of death. Therefore, inferring demographic information while studying depressive behavior from passively sensed social data can shed better light on the population-level epidemiology of depression.

The recent advancements in deep neural networks, specifically for image analysis tasks, can lead to detecting demographic features such as age and gender [29]. We aim to show that by determining and integrating a heterogeneous set of features from different modalities—aesthetic features from posted images (colorfulness, hue variance, sharpness, brightness, blurriness, naturalness), choice of profile picture (for gender, age, and facial expression), screen name, language features from both textual content and profile’s description (n-gram, emotion, sentiment), sociability from ego-network, and user engagement—we can identify individuals who are more likely to be depressed from a data set of 8,770 human-annotated Twitter users.

We address the following research questions: 1) How well does the content of posted images (colors, aesthetic, and facial presentation) reflect depressive symptoms? 2) Does the choice of profile picture show any psychological traits corresponding to a depressed online persona? 3) Are profiles pictures reliable enough to represent demographic information such as age and gender, and can they be used for community-level management of depression? 4) Are there any underlying themes among depressed individuals generated using multimodal content that can be used to reliably detect depression?

Our contributions include:

- Analysis of the content of posted images in terms of colors, aesthetic, facial presentation, and their associations with depressive symptoms;
- Uncovering the underlying relationships between visual and contextual content of likely depressed profiles obtained using a demographic inference process which can facilitate community-level management of depression; and
- Testing the performance of our interpretable heterogeneous feature set for predicting depressive symptoms.

## 1 Related work

We have divided the related work into four subsections. First, we discuss the state-of-the-art approaches for studying depressive behavior on social data. Second, we review studies that have inferred demographic information using social media data.Then, we discuss the association between color sensitivity and mental health disorders. Finally, we cover state-of-the-art studies that have used visual imagery to study individual’s behavior.

### 1.1 Mental health analysis using social media

Several efforts have attempted to automatically detect depression from social media content utilizing machine learning, deep learning, and natural language processing approaches. From conducting a retrospective study of tweets, De Choudhury *et al*., (2013) characterizes depression based on factors such as language, emotion, style, ego-network, and user engagement. They built a classifier to predict the likelihood of depression from a written post [30] or an individual’s profile [31]. Moreover, there have been significant advances due to the shared task [32] focusing on methods for identifying depressed users on Twitter at the Computational Linguistics and Clinical Psychology Workshop (CLP 2015). A corpus of nearly 1,800 Twitter users was built for evaluation, and the best models employed topic modeling [33], Linguistic Inquiry and Word Count (LIWC) features, and other metadata [34]. More recently, a neural network architecture has been introduced [35] to combine Twitter posts into a representation of users’ activities for detecting depressed users.

Another active line of research has focused on capturing warning signs of suicide and self-harm [36]. Through analysis of tweets posted by individuals attempting committing suicide, they indicate quantifiable signals of suicidal ideations. Moreover, the CLP 2016 [36] defined a shared task on detecting the severity of mental health from forum posts. *All* of these studies derive discriminative features to classify depression in user-generated content at message-level, individual-level, or community-level. The recent emergence of photo-sharing platforms such as Instagram has attracted researchers’ attention to study individual’s behavior from their visual narratives—ranging from mining their emotions [37], and happiness trend [38], to studying medical concerns [39]. Researchers have shown that people use Instagram to engage in social exchange and share their difficult experiences [13]. The role of visual imagery as a mechanism of self-disclosure by relating visual attributes to mental health disclosures on Instagram was highlighted by [14] where individual Instagram profiles were utilized to build a prediction framework for identifying markers of depression. The importance of data modality to understand user behavior on social media has been highlighted by [40]. More recently, a deep neural network sequence modeling approach that marries audio and text data modalities to analyze question-answer style interviews between an individual and an agent has been developed to study mental health [40]. Similarly, a multimodal depressive dictionary learning process was proposed to detect depressed users on Twitter [41]. They provide sparse user representations by defining a feature set consisting of social network features, user profile features, visual features, emotional features, topic-level features, and domain-specific features. Particularly, our choice to develop a multi-modal prediction framework is intended to improve upon previous work involving the use of images in multimodal depression analysis [41] and prior work on studying Instagram photos [15].

### 1.2 Demographic information inference on social media

Social media has been introduced as a critical channel to answer diverse research questions offering a wealth of data for public health research [42–44].

It can also assist in better understanding the relationship between behavioral changes and population health [45]. However, the lack of demographic indicators (e.g. age, gender, race) within the data is a major limitation for gaining deeper insights. Several research efforts have attempted to automate detection of social media users’ demographic information as summarized below. For gender inference, several studies have analyzed users’ tweets to detect gender differences reflected in linguistic patterns [46]), profile colors [47], names [48], profile images [49], social network connections [50], and user description [46]. For instance, a supervised model was developed by [51] to determine users’ gender by employing features such as screen-name, full name, profile description, and content on external resources (e.g., personal blog). Another supervised model was built to predict the user’s age group by employing features including emoticons, acronyms, slang words and phrases, punctuation, capitalization, sentence length, and included links/images, along with online behaviors such as number of friends, post time, and commenting activity [52]. To attempt to infer the age of Dutch Twitter users, a model was built that utilizes the life stage of users such as secondary school student, college student, or employee [53]. Similarly, a novel model was introduced for extracting age for Twitter users by relying on profile descriptions while devising a set of rules and patterns [54]. They also parse descriptions for occupation by consulting the SOC2010 list of occupations [55] and validating it through social surveys. A novel age inference model was developed while relying on homophily interaction information and content to predict the age of Twitter users [56]. The intuition is that people within the same age group share similar content and become friends with contemporaries. Using an extensive set of experiments, they show that their model outperformed other state-of-the-art age inference models by leveraging online interaction and content information simultaneously. The limitations of textual content for predicting age and gender was highlighted by [57]. They distinguish language use based on social gender, age identity, biological sex, and chronological age by collecting crowdsourced signals from a game in which players (crowd) guess the biological sex and age of a user based only on their tweets. Their findings indicate how linguistic markers can be misleading (e.g., a heart represented as <3 can be misinterpreted as feminine when the writer is male). Estimating age and gender from facial images by training convolutional neural networks (CNN) for face recognition is another active line of research [58].

### 1.3 Colors sensitivity and depressive behavior

The strong associations between color sensitivity and mood has been highlighted by several studies [59]. In an earlier research, a strong correlation between specific color selection such as yellow and depressive behavior has been reported by [60]. With respect to color discrimination, findings based on a sample of 20 male patients, aged 18 between 45 years old with schizophrenia and manic-depressive psychosis, indicated that when their right hemisphere was depressed, the identification of color by saturation, shade, and color tone was impaired [61]. More recently, the association of color vision with bipolar disorder explored [62]. The general findings suggest that people suffering from depression are likely to reveal their mood through their choice of colors (such as preference for darker shades) in everyday life situations [63]. In this study, we leveraged the visual content shared on Twitter for studying such signals.

### 1.4 Social media and image analysis

The recent emergence of photo-sharing platforms such as Instagram, provides a unique opportunity to study people’s behavior through the emotions [37] with broader application in personality prediction [64] and demographic inferences. Utilizing these platforms for population-levels analysis helps to improve public health concerns [39] such as obesity [65], substance use [66], depression, and anxiety [67].

With regards to personality prediction, early efforts have shown that bag-of-visual-words and Facebook profile images could predict users’ personality [68]. Various sets of features have been obtained from the images of 11,736 Facebook users were extracted to build a computational model which has more predictive power than human raters for predicting similar personality traits [69].

## 2 Dataset

This study is focused on obtaining community-level insights about depression signs and depressive behavior. As such, even though we analyzed individual’s behavioral health information–which is considered sensitive—we utilized anonymized users in our datasets as per the approved Institutional Review Board (IRB) protocol. The study was approved and the informed consent process by Wright State University Institution review Board (SC#6258) 4.1.3.

Self-disclosure refers to revealing personal and intimate information about oneself to others, which can be therapeutic for psychological well-being [70]. Previous efforts highlight diverse modes of mental health self-disclosures on social media [12]. Self-disclosure clues have been extensively utilized for creating ground-truth data for numerous social media analytic studies such as predicting users’ demographics [54], and depressive behavior [8]. For instance, vulnerable individuals may employ depressive-indicative terms in their Twitter profile descriptions. Other individuals may share their age and gender, e.g., “16 year old suicidal girl”. We employed a large dataset of 45,000 Twitter users with self-reported depressive symptoms introduced initially in [8]. All information was obtained using advanced search API [71].

To seed the search, we created a lexicon of depressive symptoms consisting of 1,500 depressive-indicative terms with the help of clinical psychologists, and employed it to collect the Twitter profiles of individuals with self-declared depressive symptoms [72]. More specifically, the dataset provides the users’ profile information including screen name, profile description, follower/followee counts, profile image, and tweet content, which can express various depression-relevant characteristics, and determine whether a user indicates any depressive behavior. Three human judges from the Department of Psychology at Wright State University assisted us in creating this annotated dataset. We reported the inter-rater agreement as K = 0.74 based on Cohen’s Kappa statistics [8]. To create a robust gold standard dataset, we discarded the instances in which at least two (out of three) of our annotators did not agree about the depressive symptoms. Our final dataset contains 8770 users with 3981 depressed users, and 4789 control users that do not express any depressive symptoms in their Twitter data. This dataset *U*_*t*_ contains the metadata values of each user such as profile descriptions, followers_count, created_at, and profile_image_url. Table 1 illustrates a sample of depressive-indicative phrases that appear in tweets from likely vulnerable users.

**Table 1:** Sample of depressive-indicative phrases collected from tweets.

Clinical Depression Symptoms	Depressive-indicative phrases in tweets
**Feeling Down**	“People hate me,” “I am Ugly,” “I am depressed”
**Sleep disorder**	“we will never sleep,” “we’re fuxx dead”
	“I’m that tired,” “why can’t I sleep”
**Lassitude**	“0 energy to do anything”
	“cba with work,” “I just want to snuggle up all day in bed”
**Obsessed with weight**	“Must not.eat,” “must.be.thin”
	“94lbs, urgh I disgust myself”
	“Obssessed with my weight,” “I just want be skinny”
**Feeling bad about yourself**	“I feel like a failure”
	“Im a piece of shix,”
**Suicidal Thought**	“I just don’t want to wake up tomorrow morning”
	“all my blades are so fuxx blunt”
	“Thinking hanging myself,” “I’ve never been so sure about suicide”
	“how much blood can bleed from a cut into a vain”

To further measure the robustness of our dataset, we conducted another experiment by obtaining additional annotation from our colleagues from the Department of Psychiatry at Weill Cornell Medical College. Using the following formula, we computed a statistically reliable sample size:

$$\begin{array}{c} SampleSize=\frac{\frac{z^{2}\times p\left(1-p\right)}{e^{2}}}{1+(\frac{z^{2}\times p\left(1-p\right)}{e^{2}N})} \end{array}$$

where N is population size, Z is z-score, e denotes margin of error, and p represents standard deviation.

Specifically, we employed our dataset of 8770 (population size), and confidence interval of 95% (margin of error 5%) to obtain 400 users as a concrete sample size. We then randomly selected 400 users from the dataset of 8770 users to be evaluated by two additional human judges (from the Department of Psychiatry at Weill Cornell Medical College) by manually annotating whether users’ content reflected depressive behavior or not. The average inter-rater agreement was (85% agreement, 0.77) based on Cohen’s Kappa statistics, which denotes substantial agreement and implies the robustness of our dataset.

### 2.1 Age enabled ground-truth dataset

**Fig 1:**
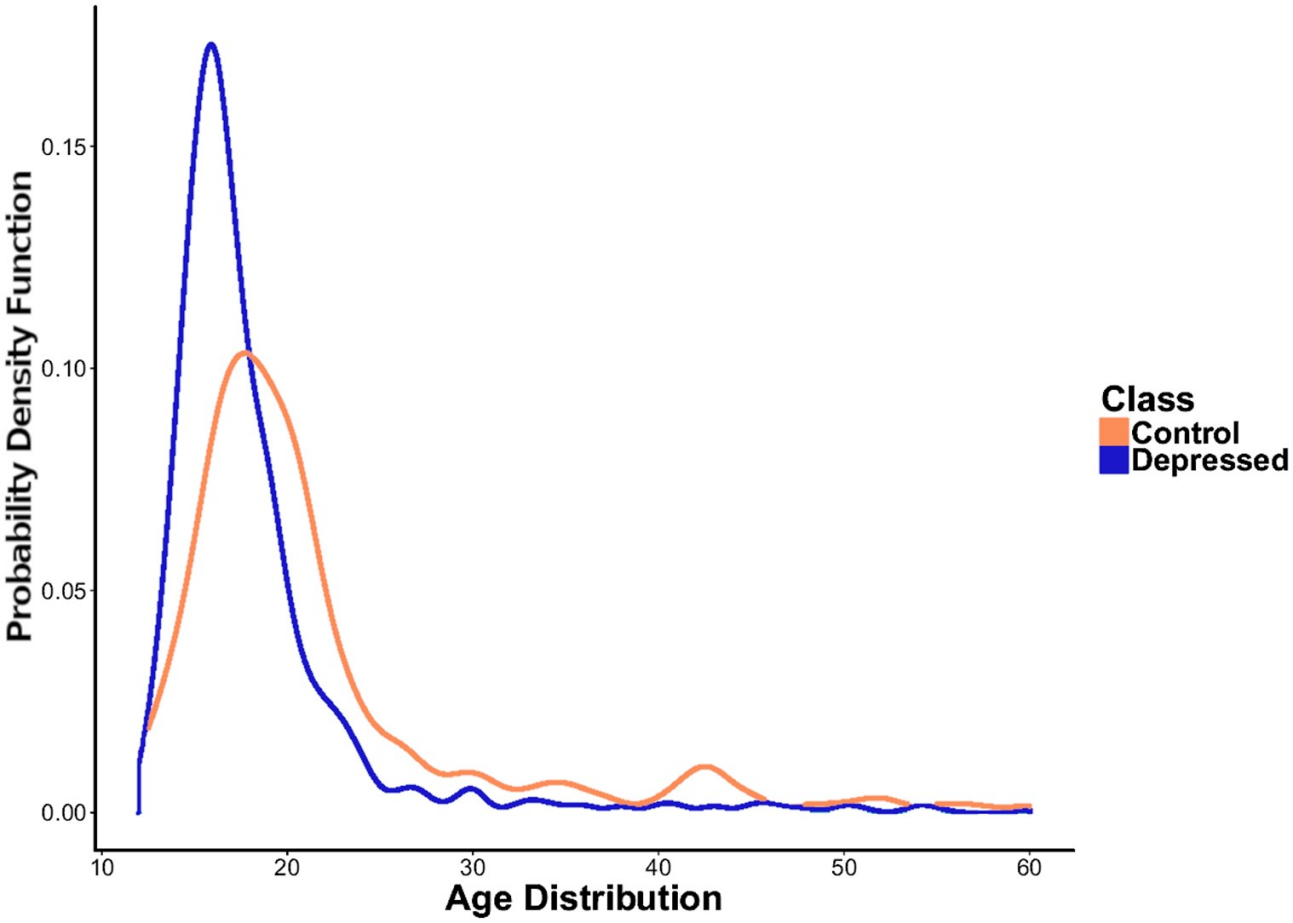
The age distribution for depressed and control users in ground-truth dataset.

We extracted a user’s age by applying regular expression patterns to profile descriptions (such as “17 years old, self-harm, anxiety, depression”) [54]. We compiled “age prefixes” and “age suffixes”, and used three age-extraction rules: 1. I am X years old, 2. Born in X, and 3. X years old, where X is a “date” or age (e.g., 1994). We selected a subset of 1061 users among *U*_*t*_ as gold standard dataset *U*_*a*_ who disclosed their age. From these 1061 users, 822 belonged to the depressed class, and 239 belonged to the control class. From the 3981 depressed users, 20.6% disclosed their age in contrast with only 4% (239/4789) among the control group, suggesting that self-disclosure of age is more prevalent among vulnerable users. Fig 1 depicts the age distribution in *U*_*a*_. The general trend, consistent with the results in [56, 73], is biased toward younger individuals. Indeed, according to the Pew Research Center, 47% of Twitter users are in general 30 years old or younger [74]. Similar data collection procedures with comparable distribution have been used previously [56]. We discuss our approach to mitigate the impact of the bias in Section 3. The median age is 17 for the depressed class versus 19 for the control class. This suggests that the depressed-user population is younger, or depressed adolescents are more likely to disclose their age in order to connect with peers (social homophily) [75].

### 2.2 Gender enabled ground-truth dataset

**Fig 2:**
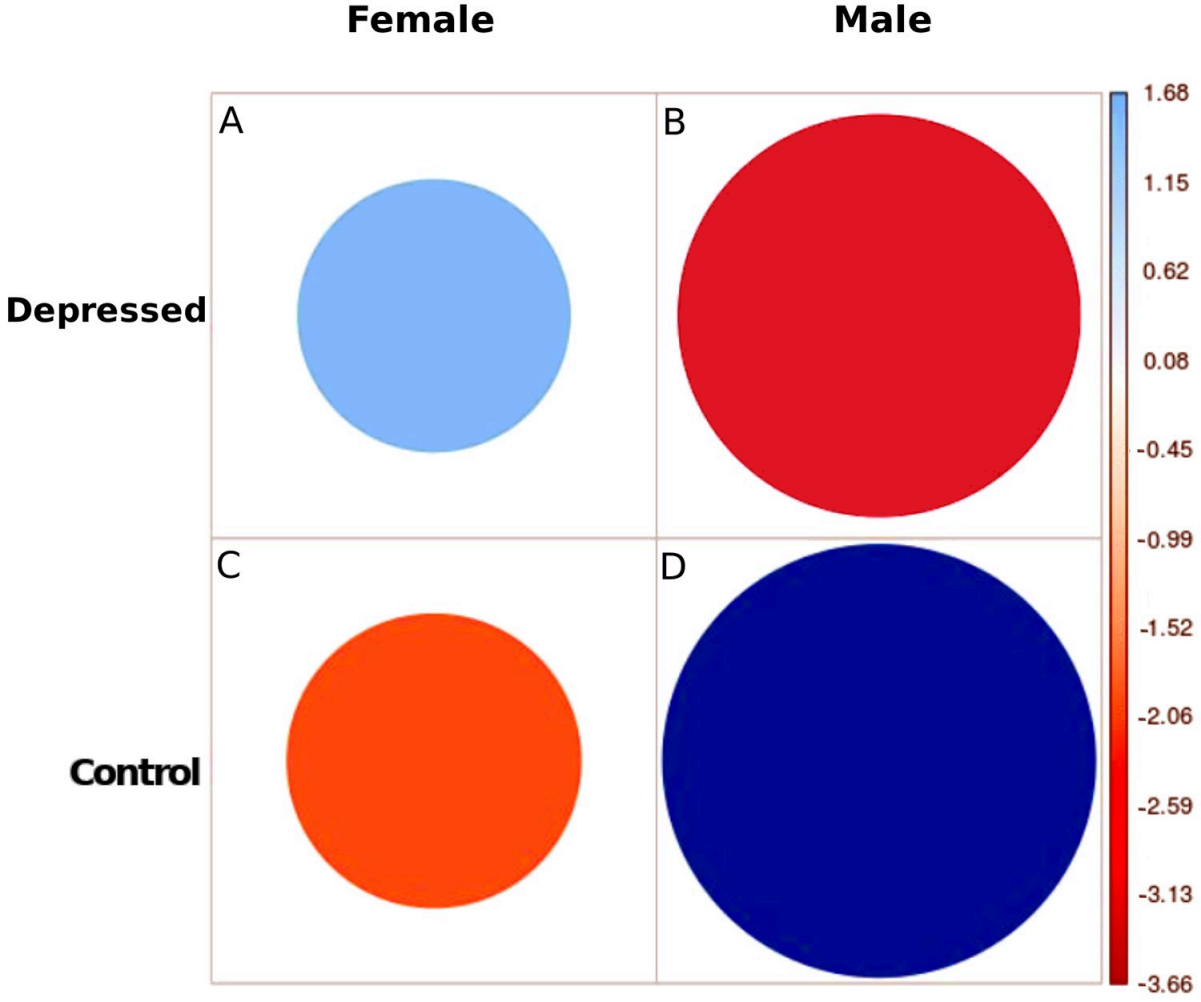
Gender and depressive behavior association (Chi-square test: Color-code: (blue:Association), (red: Repulsion), size: Amount of each cell’s contribution).

We selected a subset of 1464 users *U*_*g*_ from *U*_*t*_ who disclosed their gender in their profile description. Out of 1464 users, 64% belonged to the depressed group, and the rest (36%) belonged to the control group. 23% of the likely depressed users disclosed their gender, which is considerably higher (12%) than that of the control class. Once again, gender disclosure varies among the two gender groups. For statistical significance, we performed a chi-square test (null hypothesis: gender and depression are two independent variables). Fig 2 illustrates gender association with each of the two classes. Blue circles (positive residuals, see Fig 2A and 2D) show a positive association among corresponding row and column variables, and the red circles (negative residuals, see Fig 2B and 2C) imply a repulsion. Our findings indicate a strong association (Chi-square: 32.75, p-value:1.04e-08) between female gender, and expression of depressive symptoms on Twitter. These observations are consistent with the current literature which have shown that more women than men are diagnosed with depression [76]. In particular, the female-to-male ratio is 2:1 and 1:9 for major depressive disorder and dysthymic disorder, respectively.

## 3 Data modality analysis

We now provide an in-depth analysis of visual and textual content of vulnerable users.

### 3.1 Visual content analysis

We show that the visual content in posted images and profile images provide valuable psychological cues for understanding a user’s depression status. Profile images and posted images can surface self-stigmatization [77]. As opposed to a typical computer vision framework for object recognition that relies on thousands of predetermined low-level features, emotions reflected in facial expressions are important when assessing user’s online behavior, attributes contributing to the computational aesthetics, and sentimental quotes they may subscribe to.

The following sections present an in-depth analysis of visual content for both the depressed class and the control class with respect to three aspects: facial presence, facial expressions, and general image features.

#### 3.1.1 Facial presence

For capturing facial presence, we employed the model has been introduced in [78] where a multilevel convolutional coarse-to-fine network cascade developed to tackle facial landmark localization problem. We identified facial presentation, emotion from facial expression, and demographic features from profile images and posted images [79]. Table 2 illustrates facial presentation differences in both profile and posted images (media) for depressed users and control users in *U*_*t*_. For the control class, facial presence was significantly higher in both profile images and shared media (8%, 9% respectively) compared to the depressed class. In contrast with age and gender disclosure, vulnerable users were less likely to disclose their facial identity, possibly due to lack of confidence or fear of stigma.

**Table 2:** Facial presence comparison in profile/posted images for depressed and control users—*** alpha = 0.05.

Face_Found_in	% Of Users		*χ*^2^
	Depressed	Control	
**Media**	72%	81%	163.52***
**Profile**	4%	12%	167.2***
**Not_found**	8%	7%	2.55

#### 3.1.2 Facial expression

Following [20]’s approach, we adopted Ekman’s model [80] of six emotions: anger, disgust, fear, joy, sadness, and surprise, and used the Face++ API [79] to automatically capture these emotions from the shared images. The positive emotions were joy and surprise, and negative emotions were anger, disgust, fear, and sadness. Foreach user u in *U*_*t*_, we processed profile images and shared images for both the depressed and control groups with at least one face from the shared images (Table 3). For the images that contained multiple faces, we perform mean pooling over the frames to obtain the expected emotional features.

**Table 3:** Statistics of processed shared/profile images.

# of Processed Prof. Images		# of Processed Shared Images	
Depressed	Control	Depressed	Control
3466	4127	265785	401435

**Fig 3:**
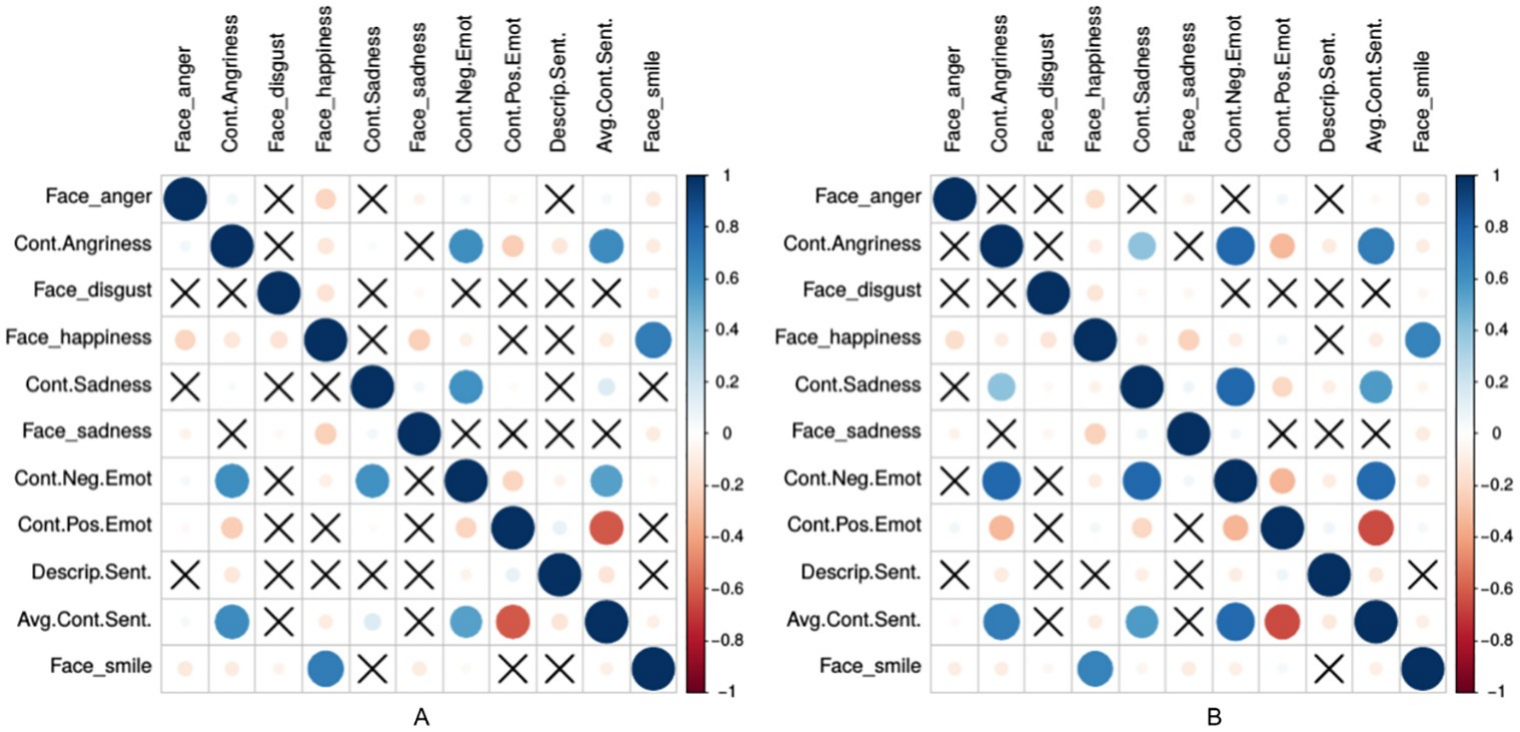
The Pearson correlation between the average emotions derived from facial expressions through the shared images and emotions from textual content for *depressed*-(a) and *control users*-(b). Pairs without statistically significant correlation are crossed (p-value <0.05).

Fig 3 illustrates the inter-correlation of these features. Additionally, we have observed that the emotions extracted from facial expressions correlated with the emotional signals captured from textual content utilizing LIWC. This indicates that visual imagery can be utilized as a complementary channel for measuring online emotional signals.

#### 3.1.3 General image features

The importance of interpretable computational aesthetic features for studying users’ online behavior has been highlighted by several efforts [81]. *Color*, as a pillar of the human vision system, has a strong association with conceptual ideas like emotion [82]. We measured the normalized red, green, blue, the mean of the original colors, brightness, and contrast relative to variations of luminance. We represented images in *Hue-Saturation-Value* color space that seems intuitive for humans, and measured the mean and variance for saturation and hue. *Saturation* is defined as the difference in intensity between different light wavelengths that compose the color. Although hue is not interpretable, high saturation indicates vividness and chromatic purity, which are more appealing to the human eye [20]. *Colorfulness* is measured as a difference against gray background [83]. *Naturalness* is a measure of correspondence between images and human perception of reality [83]. In color reproduction, *naturalness* is measured from the mental recollection of the colors of familiar objects. Additionally, there is a tendency among vulnerable users to share sentimental quotes bearing negative emotions. We performed optical character recognition (OCR) with python-tesseract [84] to extract text and their sentiment [85] score. As illustrated in Table 4, vulnerable users tend to use less colorful (higher grayscale) profile images and shared images to convey their negative feelings, and also share images that are less natural. In general, control users identified darker, grayer colors with negative mood, and generally preferred brighter, more vivid colors. By contrast, vulnerable users were found to prefer darker, grayer, and bluer colors. We found a strong positive correlation between self-declared depression and a tendency to perceive one’s surroundings as gray or lacking in color. With respect to the aesthetic quality of images (saturation, brightness, and hue), there is a significant difference between the two classes, with depressed users more frequently sharing images that are less appealing to the human eye.

**Table 4:** Statistical significance (t-statistic) of the mean of salient features for both depressed and control classes—** alpha = 0.05, *** alpha = 0.05/223.

	Feature	Depressed (*μ*)	Control (*μ*)	95 percent Conf. interval	T-stat
**Image-based**	Profile_colorfulness	108.05	118.85	(-15.38, -6.22)	-4.62***
	Profile_averageRGB	134.39	139.00	(2.3 6.92)	-3.92***
	Profile_naturalness	0.37	0.61	(-0.304, -0.192)	-12.72***
	Profile_hueVAR	0.0517	0.072	(-0.027, -0.008)	-4.56***
	Profile_saturationVAR	0.032	0.040	(-0.015, -0.003)	-3.92***
	Profile_saturationMean	0.21	0.31	(-0.122, -0.078)	-8.95***
	Shared_imageBlueChan.Mean	119.53	134.09	(-9.82, -19.28)	-6.04***
	Shared_imageGrayScaleMean	0.54	0.49	(0.03, 0.068)	5.47***
	Shared_imageColorfulness	106.12	122.37	(-14.98, -10.753)	-11.94***
	Shared_imageSaturationVAR	0.033	0.047	(-0.01, -0.010)	-9.26***
	Shared_imageSaturationMean	0.198	0.289	(-0.106, -0.074)	-10.95***
	Shared_imageNaturalness	0.486	0.651	(-0.193, -0.136)	-16.28***
**Social-based**	Friends_count	610.196	1380.25	(-1023, -516)	-5.98***
	Followers_count	589.47	1340.83	(-1148.08, -354)	-3.727**
	Statuses_count	3722	7766	(-6281, -1806)	-3.55**
	Avg_tweet_favorite_count	0.22	0.67	(-0.781, -0.103)	-2.57**
	Avg_tweet_retweet_count	876.75	2720	(-2673, -1013)	-4.36***
	Favourites_count	2021	5199.67	(-5038, -1317)	-3.35**

We employed an independent samples t-test, while adopting Bonferroni Correction as a conservative approach to adjust the confidence intervals. Overall, we had 223 features, and chose Bonferroni-corrected *alpha* level of 0.05/223 = 2.24*e* − 4 (*** *p* < *alpha*, **p < 0.05).

In general, the control users identified darker, grayer colors with negative moods, and generally preferred brighter, more vivid colors. In contrast, vulnerable users preferred darker, grayer colors, and bluer images. Vulnerable users shared images that are less aesthetically pleasing with lower sharpness, and those that do not contain faces or contain only one face. On the other hand, control users tended to use sharper images with multiple faces. Additionally, vulnerable users shared images with more text content, often containing depressive quotes and negative sentiments.

The desire to socialize and connect with others is also manifested in the visual imagery of vulnerable users. The images shared by vulnerable users tend to contain a single face (belonging to the user), rather than surrounded by friends and family. This further indicates the focus on the self, which is one of the most consistent markers of a mental disorder. This is also associated with an extensive usage of first person singular pronouns—which is another reliable marker of depression in content analysis of depressive behavior.

### 3.2 Demographics inference & language cues

LIWC [86] has been used extensively for examining the latent dimensions of self-expression for analyzing personality [87], depressive behavior, demographic differences [53, 57], etc. Several studies have shown that females employ more first-person singular pronouns [88], and deictic language (context-dependent words) [89], while males tend to use more articles [90] which characterize concrete thinking, and formal, informational, affirmative words [91]. For age analysis, the salient findings show that older individuals use more future tense verbs, [88] suggesting a shift in focus while aging. They also show more positive emotions [92], employ fewer self-references (i.e. ‘I’, ‘me’), and more first person plural pronouns [88]. Depressed users employ first person pronouns more frequently [93], and repeatedly use negative emotions and anger words. We analyzed psycholinguistic cues and language style to study the association between depressive behavior and demographics. Specifically, we adopted Levinson’s adult development grouping [94] that partitions users in *U*_*a*_ into 5 age groups: (14,19], (19,23], (23,34], (34,46], and (46,60]. Then, we applied LIWC for characterizing linguistic styles for each age group for users in *U*_*a*_.

#### 3.2.1 Qualitative language analysis

The recent LIWC version [86] summarizes textual content in terms of language variables such as analytical thinking, clout, authenticity, and emotional tone. It also measures other linguistic dimensions such as descriptor categories (e.g., percent of target words gleaned from the dictionary, or words longer than six letters—Sixltr), informal language markers (e.g., swear words, netspeak), and other linguistic aspects (e.g., first person singular pronouns).

**Thinking Style:** The words we use to communicate can reveal our style of thinking. There are two common approaches for extracting an individual’s thinking style. First, measuring one’s natural way of trying to understand, analyze, and organize complex events has a strong association with analytical, formal, and logical thinking. LIWC relates higher analytic thinking to more formal and logical reasoning, whereas a lower value indicates a focus on narratives. Second, cognitive processing, which measures problem solving in the mind, is captured through words such as “think,” “believe,” “realize,” and “know” and demonstrates “certainty” in communication. High values for analytical thinking implies clarity of thought.

Critical thinking ability is related to education [95], and is impacted by different stages of cognitive development at different ages [96]. It has been shown that older people communicate with greater cognitive complexity while comprehending nuances and subtle differences [95]. All of these findings corroborate with our results (Table 5).

**Table 5:** Statistical significance test of linguistic patterns/visual attributes for different age groups with one-way ANOVA, *** alpha = 0.001, ** alpha = 0.01.

	Feature	Mean (SD)					F-value
		[11,19)	[19,23)	[23,34)	[34,46)	[46,60)	
**Text-based**	**Analytic**	27.62 (16.62)	38.61 (19.16)	47.28 (20.69)	67.88 (18.51)	72.05 (20.79)	84***
	**Authentic**	58.54 (19.54)	55.04 (20.04)	49.21 (22.05)	33.99 (19.73)	28.39 (19.04)	22***
	**Clout**	51.6 (21.35)	53.43 (21.26)	56.27 (19.81)	70.28 (17.46)	71.21 (13.50)	9***
	**Dic**	85.04 (6.06)	82.63 (6.21)	80.48 (6.56)	75.87 (6.91)	74.09 (5.95)	37***
	**Article**	3.52 (0.78)	3.92 (0.73)	4.00 (0.80)	4.52 (1.38)	5.13 (1.00)	35***
	**Sixltr**	15.48 (2.84)	16.58 (3.07)	18.65 (3.71)	20.88 (4.74)	21.33 (4.11)	52***
	**Cogn. words**	12.17 (2.53)	11.24 (2.38)	10.99 (2.55)	8.36 (2.63)	8.75 (1.96)	28***
	**Self-ref**	14.13 (2.35)	12.45 (2.56)	10.96 (2.60)	9.05 (3.69)	7.55 (3.38)	85***
	**Swear**	0.96 (0.59)	0.89 (0.53)	0.57 (0.48)	0.36 (0.41)	0.33 (0.30)	18***
	**Money**	0.27 (0.40)	0.38 (0.19)	0.45 (0.25)	0.52 (0.22)	0.78 (0.37)	15***
	**Work**	0.80 (0.39)	1.09 (0.53)	1.31 (0.76)	1.67 (0.83)	2.02 (1.01)	69***
**Image-based**	**Profile_Naturalness**	37.80 (13.84)	48.05 (18.64)	52.33 (28.51)	64.33 (24.53)	68.07 (15.28)	10***
	**Profile_SaturationMean**	20.31 (1.95)	23.27 (1.99)	29.78 (1.99)	38.76 (2.14)	33.13 (1.94)	9***
	**Profile_Colorfullness**	106.47 (42.70)	107.95 (39.15)	111.01 (42.09)	113.97 (35.48)	123.60 (27.60)	0.89
	**Shared_avgRGB**	139.20 (18.12)	140.45 (16.00)	131.55 (16.32)	133.74 (22.41)	139.02 (22.30)	3**
	**Profile_GrayMean**	0.471 (0.19)	0.474 (0.16)	0.456 (0.21)	0.470 (0.14)	0.450 (0.11)	0.12

**Fig 4:**
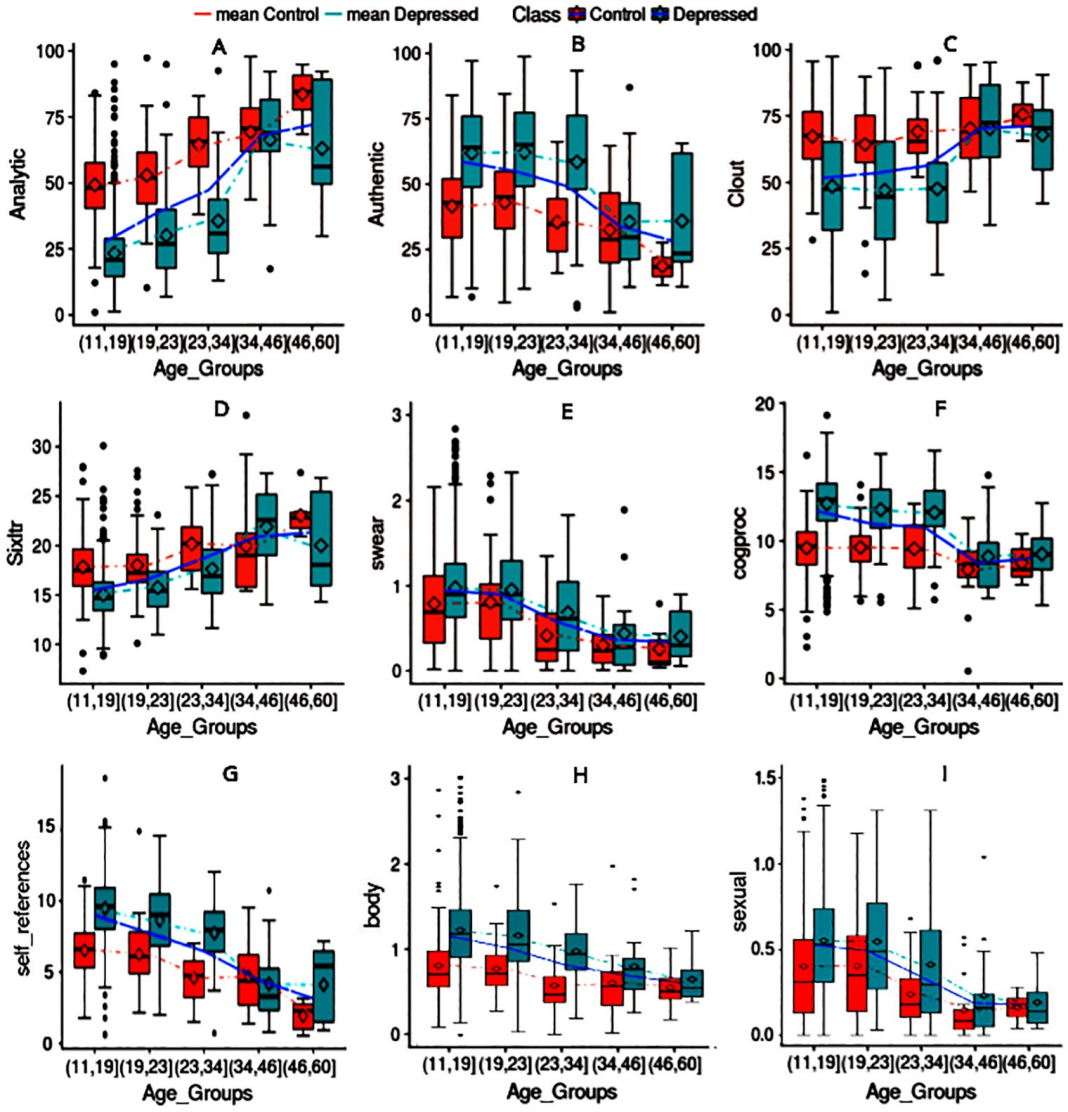
Characterizing linguistic patterns in two aspects: Depressive-behavior and age distribution.

We observed notable differences in raw intelligence and the ability to think analytically in depressed and control users among different age groups (see Fig 4A and 4F and Table 5). Overall, vulnerable younger users do not think as logically based on their relative analytical score and cognitive processing ability. We can also observe that the differences between age groups above 35 tend to become smaller [97].

**Authenticity:** Authenticity measures the degree of honesty. Authenticity is often assessed by measuring present tense verbs, first person singular pronouns (e.g., I, me, my), and by examining the linguistic manifestations of false stories [98]. People who lie use fewer self-references, and fewer complex words. Psychologists often see a child’s first successful lie as a mental milestone growth [99]. There is a decreasing trend in authenticity with age (see Fig 4B). Authenticity for depressed adolescents is strikingly higher than their control peers, and decreases with age (Fig 4B).

**Clout:** People with high clout speak more confidently and with certainty, employing more social words with fewer negations (e.g., no, not) and swear words. In general, mid-life is relatively stable w.r.t. relationships and work. A recent study has shown that age 60 is best for self-esteem [100] as people take on managerial roles at work, and maintain satisfyinging relationships with their spouses. We see the same pattern in our data (see Fig 4C and Table 5). Unsurprisingly, lack of confidence (the 6th PHQ-9 [101] symptom) is a distinguishable characteristic of vulnerable users, leading to their lower clout scores, especially among depressed users younger than 34 years old.

**Self-references:** First person singular words often indicate interpersonal involvement, and their high usage is associated with negative affective states such as nervousness and depression [92]. Consistent with prior studies, the frequency of first person singular words for depressed users is significantly higher compared to that of the control class. Similarly to [92], adolescents tend to use more first-person (e.g. I), and second person singular (e.g. you) pronouns (Fig 4G). The impact of the above phenomenon is reflected in significantly higher frequency of self-references for depressed adolescents. As with the control class, a downtrend suggests that as depressed individuals age, they make more distinctions and psychologically distance themselves from their topics.

**Informal Language Markers; Swear, Netspeak:** Swear lexicon includes terms such as “fu**”, “dam*”, and “shi*”. Several studies have highlighted that the use of profanity by young adults has significantly increased over the last decade [102]. We observed the same pattern in both the depressed and the control classes (Table 5), with a higher rate for depressed users [10]. Psychologists have also shown that swearing may indicate that an individual is not a fragmented member of a society [103]. Depressed adolescents who show a higher rate of interpersonal involvement and relationships, have a higher rate of cursing (Fig 4E). Also, Netspeak lexicon measures the frequency of terms such as ‘lol’ and ‘thx’. Although the rate is higher for the depressed class, we did not find any pattern concerning adult development.

**Sexual, Body:** The sexual lexicon contains terms like “horny”, “love”, and “incest”, and body terms like “ache”, “heart”, and “cough”. Both start with a higher rate for depressed users and decreases gradually as they age, possibly due to changes in sexual desire with age [104] (Fig 4H and 4I and Table 5).

#### 3.2.2 Quantitative language analysis

We employed a one-way ANOVA to compare the impact of various factors, and validate our findings above. Table 5 illustrates our findings, with a degree of freedom (df) of 1055. The null hypothesis is that the sample means for each age group are similar for each of the LIWC features.

### 3.3 Demographic prediction

We leveraged both the visual and textual content for predicting age and gender.

#### 3.3.1 Prediction with textual content

We employed [105]’s weighted lexicon of terms that uses the dataset of 75,394 Facebook users who shared their status, age, and gender. The predictive power of this lexica was evaluated on Twitter, and Facebook, showing promising results [105]. Utilizing these two weighted lexicon of terms, we are predicting the demographic information (age or gender) of *user*_*i*_ (denoted by *Demo*_*i*_) using the following equation:

$$\begin{array}{c} Demo_{i}=\sum_{terms\epsilon lex}Weight_{lex}\left(term\right)*\frac{Freq{\left(term,doc\right)}_{i}}{WC{\left(doc\right)}_{i}} \end{array}$$

where *Weight*_*lex*_(*term*) is the lexicon weight of the term, and *Freq*(*term*, *doc*)_*i*_ represents the frequency of the term in the user generated *doc*_*i*_, and *WC*(*doc*)_*i*_ measures total word count in (*doc*)_*i*_. As our data are biased toward younger individuals, we report age prediction performance for each age group, separately (Table 6). Moreover, to measure the average accuracy of this model, we built a balanced dataset (keeping the total number of users above 23—416), and then randomly sampled the same number of users from the age ranges (11,19] and (19,23]. The average accuracy of this model was 0.63 for depressed users, and 0.64 for the control class. Table 8 illustrates the performance of gender prediction for each class. The average accuracy was 0.82 on *U*_*g*_ ground-truth dataset.

**Table 6:** Age Prediction performance from visual and textual content for different age group(years old).

Group	Measure	Text-based				Image-based (Profile)				Image-based (Media)			
		(11,19]	(19,23]	(23,34]	(34,46]	(11,19]	(19,23]	(23,34]	(34,46]	(11,19]	(19,23]	(23,34]	(34,46]
**Depressed**	**Sensitivity**	0.23	0.38	0.65	0.33	0.29	0.29	0.22	1.0	0.11	0.1	0.19	0.22
	**Specificity**	0.95	0.53	0.69	0.96	0.92	0.92	0.57	0.80	0.96	0.94	0.72	0.58
	**ACC**	0.59	0.46	0.67	0.65	0.47	0.46	0.40	0.900	0.50	0.49	0.46	0.40
**Control**	**Sensitivity**	0.14	0.31	0.62	0.69	0.12	0.1	0.40	0.25	0.15	0.30	0.63	0.64
	**Specificity**	0.98	0.63	0.61	0.90	0.90	0.95	0.53	0.75	0.98	0.62	0.60	0.91
	**ACC**	0.56	0.47	0.62	0.80	0.49	0.48	0.47	0.51	0.56	0.46	0.62	0.77

#### 3.3.2 Prediction with visual imagery

Inspired by [78]’s approach for facial landmark localization, we used their pre-trained CNN consisting of convolutional layers, including unshared and fully-connected layers, to predict gender and age from both the *profile* and *shared images*. We evaluated the performance of the gender and age prediction task on *U*_*g*_ and *U*_*a*_, respectively, as shown in Table 6.

#### 3.3.3 Demographic prediction analysis

We delved deeper into the benefits and drawbacks of each data modality for demographic information prediction. This is crucial as the differences between language cues between age groups above 35 tend to become smaller (see Fig 4A, 4B and 4C), making the prediction harder for older individuals [97]. In this case, the other data modality (e.g., visual content) played an integral role as a complementary source for age inference. For gender prediction, on average, the profile image-based predictor provided a more accurate prediction for both the depressed and the control class (0.92 and 0.90), compared to the content-based predictor (0.82). For age prediction (see Table 6), the textual content-based predictor (on average 0.60) outperformed both of the visual-based predictors (on average profile: 0.51, Media: 0.53). However, not every user provided facial identity on his or her account (see Table 2). We studied facial presentation for each age group to examine any association between age group, facial presentation, and depressive behavior (see Table 7). We can see youngsters in both the depressed and control classes are not likely to present their face in their profile image. Less than 3% of vulnerable users between 11-19 years revealed their facial identity. Although the content-based gender predictor was not as accurate as the image-based predictor, it is adequate for population-level analysis (see Table 8).

**Table 7:** Facial presentation distribution for different age group(in years old) in profile and media.

	% Users Faces_Found_ in_Profile					% Users Faces_Found_ in_Media				
	[11,19)	[19,23)	[23,34)	[34,46)	[46,60)	[11,19)	[19,23)	[23,34)	[34,46)	[46,60)
**Control**	4.55	9.58	13.84	17.85	21.42	89.70	88.35	78.46	67.85	78.57
**Depressed**	2.71	5.88	10.52	8.33	14.28	90.21	90.58	76.31	83.33	85.71

**Table 8:** Gender prediction performance through visual and textual content.

Face found in	Image-based Predictor						Content-based Predictor					
	Depressed			Control			Depressed			Control		
	Sens.	Spec.	ACC (95% CI)	Sens.	Spec.	ACC (95% CI)	Sens.	Spec.	ACC (95% CI)	Sens.	Spec.	ACC (95% CI)
**Profile**	0.90	1.0	0.92 (0.80, 0.98)	0.91	0.87	0.90 (0.81, 0.95)	0.87	0.50	0.82 (0.79, 0.85)	0.86	0.76	0.82 (0.79, 0.85)
**Media**	0.57	0.70	0.58 (0.54, 0.62)	0.46	0.65	0.51 (0.46, 0.55)						

## 4 Multi-modal prediction framework

**Fig 5:**
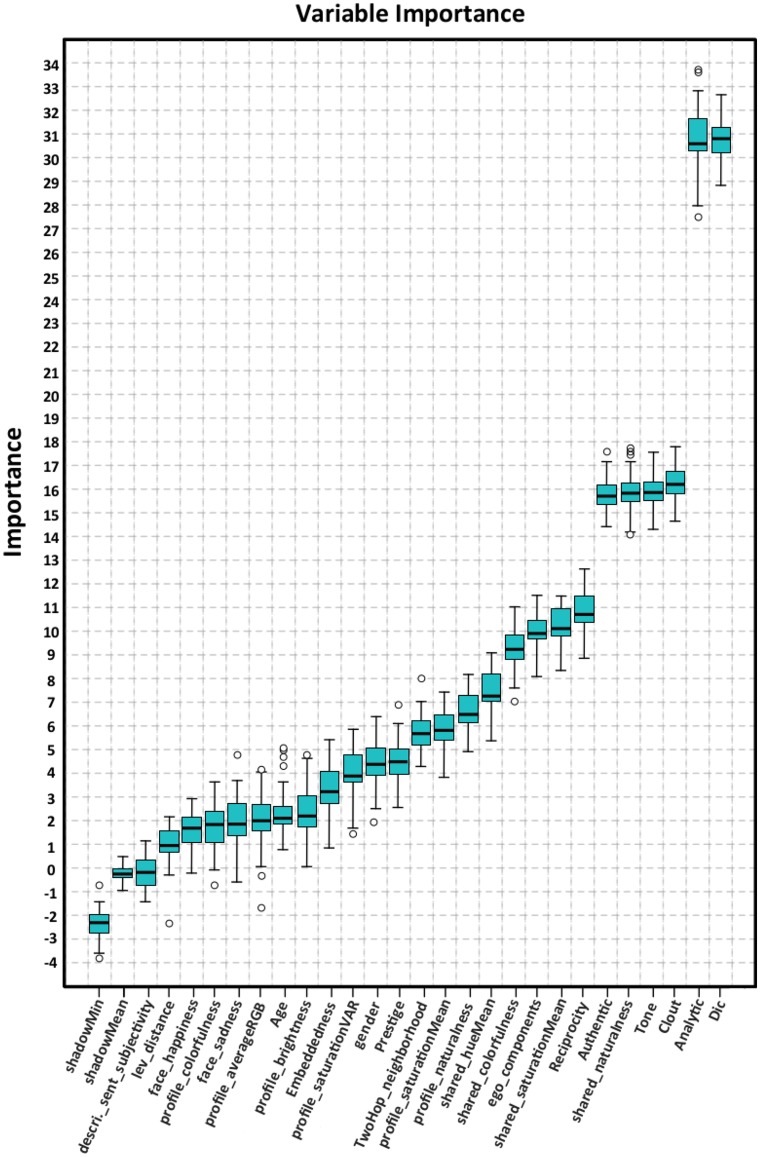
Ranking features obtained from different modalities with an ensemble algorithm.

We used the above findings for predicting depressive behaviors. Our model exploits an early fusion [40] technique in feature space and requires modeling each user *u* in *U*_*t*_ as vector concatenation of individual modality features. As opposed to the computationally expensive late fusion schemes, where each modality requires a separate supervised modeling, this model reduces the learning effort and has shown promising results [106]. To develop a generalizable model that avoids overfitting, we performed feature selection using statistical tests and *all relevant* ensemble learning models. Adding feature selection tests adds randomness to the data by creating shuffled copies of all features (shadow feature), and then trains the Random Forest classifier on the extended data. Iteratively, it checks whether the actual feature has a higher Z-score than its shadow feature (See Algorithm 1 and Fig 5) [107].

**Algorithm 1**: **Ensemble Feature Selection**

**Function** *Main*

** for** each Feature *X*_*j*_ ∈ *X* **do**

  *ShadowFeatures* ← *RndPerm*(*X*_*j*_)

 *RndForrest*(*ShadowFeatures*, *X*);

  *Calculate Imp* (*X*_*j*_, *MaxImp*(*ShadowFeatures*));

 **if** *Imp*(*X*_*j*_) > *MaxImp*(*ShadowFeatures*) **then**

  *Generate next hypothesis*, **return** *X*_*j*_

 *Once all hypothesis generated*;

 *Perform* Statistical Test

 $H_{0}:H_{i}=E\left(H\right)vsH_{1}:H_{i}\neq E\left(H\right)\;H_{i}∼N\left(\left(0.5N\right)\left({\left(\sqrt{0.25N}\right)}^{2}\right)\right)$ *//Binomial Distribution*;

 **if** *H*_*i*_ ≫ *E*(*H*) **then**

  *Feature is important*

 **else**

  *Feature is important*

Next, we adopted an ensemble learning method which integrated the predictive power of multiple learners with two main advantages; a high degree of interpretability with respect to the contributions of each feature, and a high predictive power. For prediction, we have $y_{i}^{\prime }=\sum_{t=1}^{m}f_{t}\left(u_{i}\right)$ where *f*_*t*_ is a weak learner and $y_{i}^{\prime }$ denotes the final prediction.

In particular, we optimized the loss function:

$$\begin{array}{c} L^{<t>}=\sum_{i=1}^{n}l\left(y_{i},y_{i}^{{}^{\prime }<t-1>}+f_{t}\left(u_{i}\right)\right)+\varphi \left(f_{t}\right) \end{array}$$

where *φ* incorporates *L*1 and *L*2 regularization. In each iteration, the new *f*_*t*_(*u*_*i*_) is obtained by fitting the weak learner to the negative gradient of loss function. Particularly, by estimating the loss function with Taylor expansion:

$$\begin{array}{c} L^{<t>}∼\sum_{i=1}^{n}l\left(y_{i},y_{i}^{{}^{\prime }<t-1>}\right)+\left(\frac{\partial l(y_{i},y_{i}^{{}^{\prime }<t-1>}}{\partial y_{i}^{{}^{\prime }<t-1>}}\right)f_{t}\left(u\right)+\left(\frac{\partial^{2}l(y_{i},y_{i}^{{}^{\prime }<t-1>}}{\partial y_{i}^{{}^{\prime }<t-1{>}^{2}}}\right)f_{t}{\left(u_{i}\right)}^{2}\; \end{array}$$

where its first expression is constant, the second and the third expressions are first (*g*_*i*_) and second order derivatives (*h*_*i*_) of the loss.

$$\begin{array}{c} L^{<t>}=\sum_{i=1}^{n}\left(g_{i}f_{t}\left(u_{i}\right)+h_{i}f_{t}\left(u_{i}\right)\right)+\varphi \left(f_{t}\right) \end{array}$$

To explore the weak learners, assume *f*_*t*_ has k leaf nodes, *I*_*j*_ be subset of users from *U*_*t*_ belongs to the node *j*, and *w*_*j*_ denotes the prediction for node *j*. Then, for each user *i* belonging to *I*_*j*_, *f*_*t*_(*u*_*i*_) = *w*_*j*_ and $\varphi \left(f_{t}\right)=1/2\lambda \sum_{j=1}^{k}W_{j}^{2}+\gamma k$

$$\begin{array}{c} L^{<t>}=\sum_{j=1}^{k}\left[\left(\sum_{i\epsilon I_{j}}g_{i}\right)w_{j}+1/2\left(\sum_{i\epsilon I_{j}}h_{i}+\lambda \right)w_{j}^{2})\right]+\gamma k \end{array}$$

**Fig 6:**
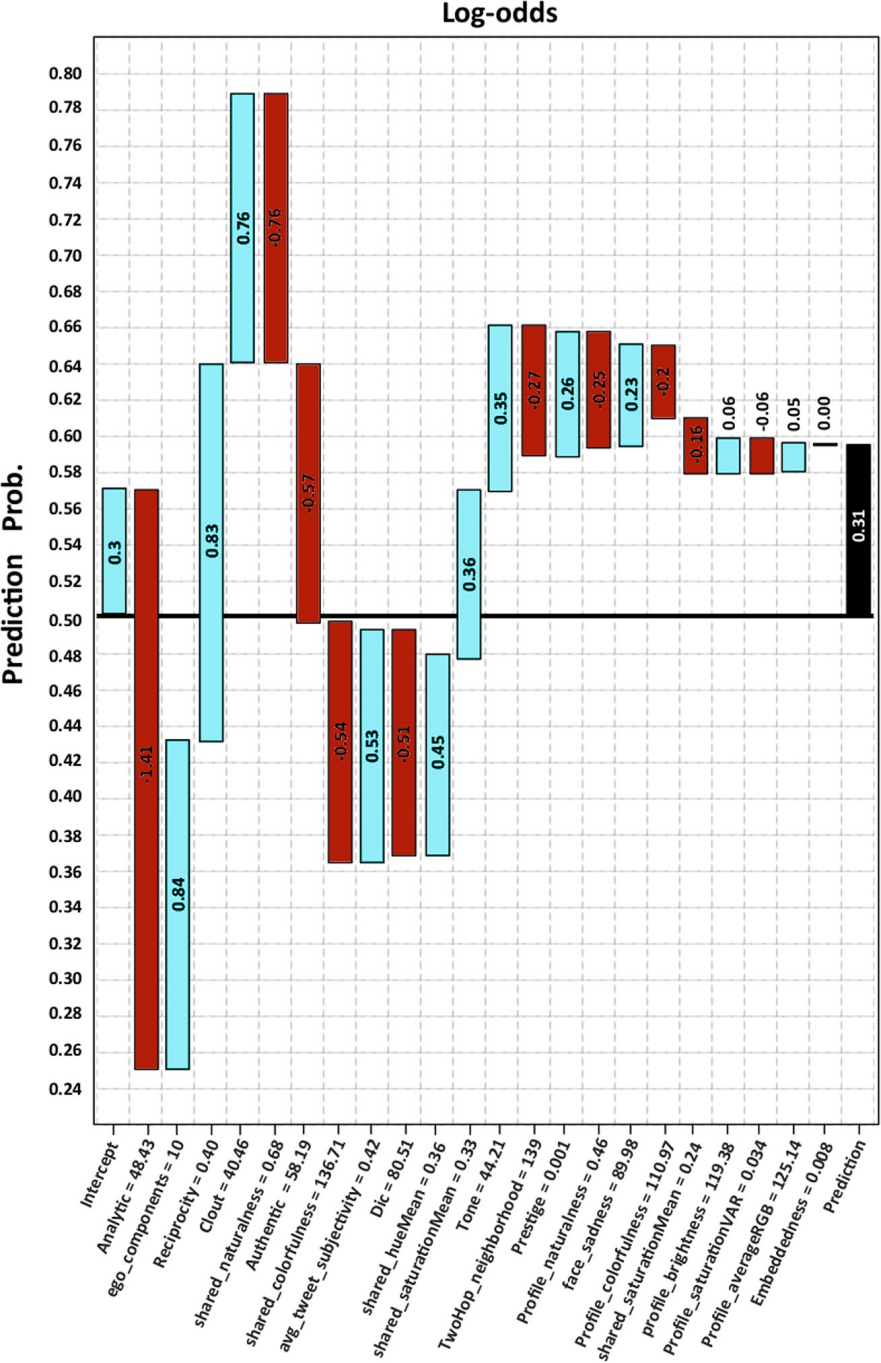
The explanation of the log-odds prediction of outcome (0.31) for a sample user (y-axis shows the outcome probability (depressed or control), the bar labels indicate the log-odds impact of each feature).

Next, for each leaf node *j*, deriving w.r.t *w*_*j*_:

$$\begin{array}{c} w_{j}=\frac{-\sum_{i\epsilon I_{i}}g_{i}}{\sum_{i\epsilon I_{j}}h_{i}+\lambda } \end{array}$$

and by substituting weights:

$$\begin{array}{c} L^{<t>}=-1/2\sum_{j=1}^{k}\frac{{\left(\sum_{i\epsilon I_{j}}g_{i}\right)}^{2}}{\sum_{i\epsilon I_{j}}h_{i}+\lambda }+\gamma k \end{array}$$

which represents the loss of fixed weak learners with *k* nodes. The trees are built sequentially, such that each subsequent tree aims to reduce the errors of its predecessor trees. Although, the weak learners have a higher degree of biases, the ensemble model produces a strong learner that effectively integrates the weak learners by reducing bias and variance (the ultimate goal of supervised models) [108, 109]. Table 9 illustrates how our multimodal framework outperforms the baselines for identifying depressed users in terms of average specificity, sensitivity, F-Measure, and accuracy in a 10-fold cross-validation setting on *U*_*t*_ dataset. Fig 6 shows how the likelihood of being classified into the depressed class varies with each feature added to the model for a sample user in the dataset. The prediction bar (the black bar) shows that the log-odds of prediction is 0.31, that is, the likelihood of this person being a depressed user is 57% (1 / (1 + exp(-0.3))). The figure also sheds light on the impact of each contributing feature. The waterfall charts represent how the probability of being depressed varies when adding each feature. For example, for our dataset, the *“Analytic thinking”* score measured by LIWC from the tweet content is a high value of 48.43 (Median:36.95, Mean: 40.18) and this decreases the chance of the user being classified into the depressed group by the log-odds of -1.41. This is due to the fact that depressed users have significantly lower *“Analytic thinking”* scores compared to the control class. Moreover, the *“Clout”* score of 40.46 is considered a low value (Median: 62.22, Mean: 57.17), and increases the chance of being classified as a depressed user. This is justifiable given the clear association between low self-esteem and risk for depression. With respect to the visual features, the mean and the median of *“shared colorfulness”* is 112.03 and 113, respectively. The value of 136.71 would be high, and decreases the chance of being depressed by log-odds of -0.54. As mentioned earlier, depressed users preferred darker, and grayer colors. The score of 0.46 as *“profile naturalness”* is considered high compared to 0.36 (the mean for the depressed class) which justifies pull down of the log-odds by −0.25. Using network features, for instance, the *“two hop neighborhood”* for depressed users (Mean: 84) are less than that of the control users (Mean: 154), and is reflected in pulling down the log-odds by -0.27.

**Table 9:** Model’s performance for depressed user identification in Twitter using different data modalities.

Model#	Data Source	Ref.	Year	Features					Model	Spec.	Sens.	F-1	Acc.
				N-grams	LIWC	Sentiment	Topics	Metadata					
I	Content	[110]	2016	X					NB	0.69	0.70	0.69	0.70
II		[111]	2016	X		X		User Acti.	N/A (LR)	0.73	0.74	0.73	0.74
III		[112]	2015	X	X	X		User Acti.	Log-linear	0.83	0.80	0.81	0.82
IV		[113]	2015	X	X	X	X		LR	0.84	0.83	0.84	0.84
V		[114]	2015	X	X	X	X	User Acti.	SVM	0.86	0.84	0.85	0.85
VI		N/A	N/A	X					SVM(Pre. embed.)	0.72	0.72	0.72	0.72
VII		N/A	N/A	X					SVM(Train w2vec)	0.70	0.70	0.70	0.70
VIII	Cont., Net.	[10]	2013	X	X	X			SVM, PCA	0.84	0.80	0.83	0.85
IX	Image	N/A	N/A	N/A					LR	0.68	0.67	0.67	0.68
X		N/A	N/A						SVM	0.69	0.67	0.67	0.69
XI		N/A	N/A						RF	0.72	0.70	0.69	0.71
**Ours**	Cont.,Image,Net.	N/A	X	X	X	X	X	X	N/A	**0.87**	**0.92**	**0.90**	**0.90**

### 4.1 Baselines

To test the efficacy of our multi-modal framework for detecting depressed users, we compared it against existing content, content-network, and image-based models (based on the aforementioned general image features, facial presence, and facial expressions).

#### 4.1.1 Content-based models

**Fig 7:**
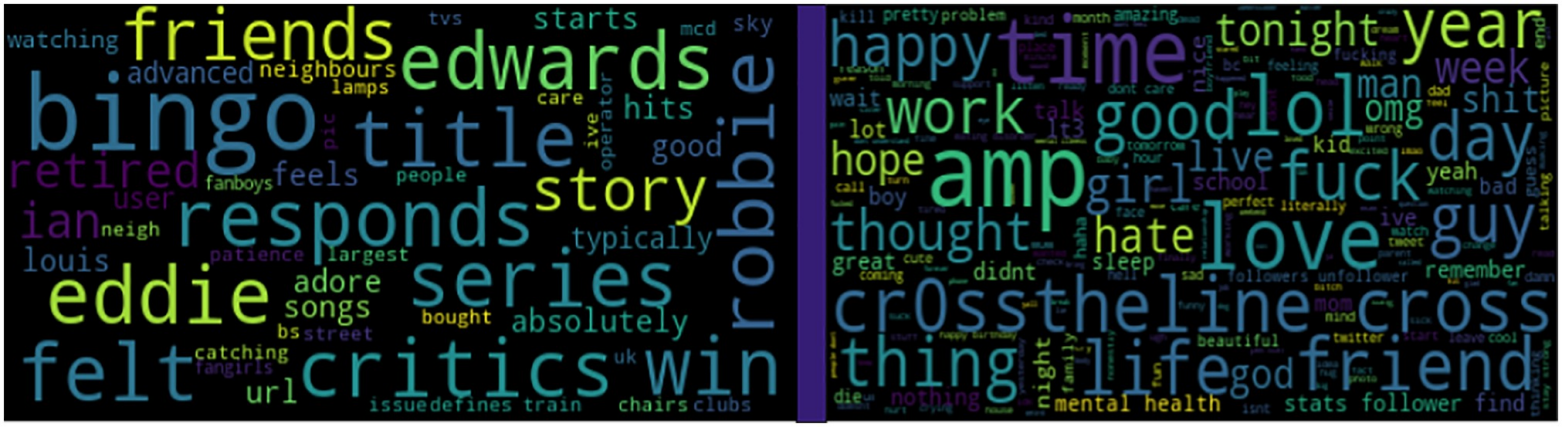
Word usage difference of likely vulnerable individuals versus random profiles.

Language biases in social media posts can be a good representative of emotional state. Fig 7 illustrates the word clouds that distinguish the word usage of likely-depressed and non-depressed profiles. It is clear that depressed users often care more about their appearance. This is indicative by their usage of terms such as “pretty” and “beautiful.” They also have a tendency to talk about their family and relations using words such as *family*, *hugs*, *parents*, *daddy*, *mums*, *sigh*, *grandma*, *maam*, *friendless*, *love*, *friend*, *mommy*, *people*, *boyf*, and *gf*. In contrast, the control users usually talk about daily events and news such as “hurricane” and “Trump”. Such differences in word usage highlight the fact that user generated words can be distinguishable features for detecting depressed user profiles. See Table 9 for the comparative performance of our prediction framework against state-of-the-art methods used for predicting depressive behaviors—many of which employed the same feature sets and hyperparameter settings (see Models I-V). Several prior efforts have demonstrated that word embedding models can reliably enhance short text classification [115], Model VI by employing pre-trained word embeddings which have trained over 400 million tweets [116] while representing a user with retrieving word vectors for all the words a user used in tweets and profile description. We aggregate these word vectors through their means and feed it as input to a SVM classifier with a linear kernel. In Model VII, we employed [8]’s dataset of 45,000 self-reported depressed users and trained a Skip-gram model with negative sampling to learn word representations. We chose this model because it generates robust word embeddings even when the collection of training words are sparse [117]. We set dimensionality to 300 and a negative sampling rate to 10 sample words, which has shown promising results with medium-sized datasets [117]. Besides, we have observed that many vulnerable users chose specific account names, such as “Suicidal_Thoughxxx,” and “younganxietyyxxx,” which are good indicators of their depressive behavior. We used Levenshtein distance between depression indicative terms in [8]’s depression lexicon and the screen name to capture their degree of semantic similarity [118].

#### 4.1.2 Image-based models

We employed the aforementioned visual content features including facial presence, aesthetic features, and facial expression for depression prediction. We use three different models: Logistic Regression (Model IX), SVM (Model X), and Random Forest (Model XI). The poor performance of image-based models suggests that relying on a unique modality would not be sufficient for building a robust model due to the complexity and abstruse nature of the prediction task.

#### 4.1.3 Network-based models

Network-based features indicate the user’s desire to socialize and connect with others. There is a notable difference between the number of friends, followers, favorites, and status count for depressed and control users (see Table 4). For building a baseline Model VIII, we obtained egocentric network measures for each user based on the network formed using @-replies interactions among them. The egocentric social graph of a user u is an undirected graph of nodes in u’s two-hop neighborhood in our *U*_*a*_ dataset, where the edge between nodes u and v implies that there has been at least one @-reply exchange. Network-based features including *Reciprocity*, *Prestige Ratio*, *Graph Density*, *Clustering Coefficient*, *Embeddedness*, *Ego components* and *Size of two-hop neighborhood* were extracted from each user’s network [10] to reliably capture user context for depression prediction.

High values for the three metrics—clustering coefficient, embeddedness, and number of ego networks—indicates that the depressed users tend to build a close network of trusted people to share their mental health issues. For both graph density and size of the two-hop neighborhood, a lower value indicates fewer interactions.

## Conclusion and future work

We presented an in-depth analysis of visual and contextual content of likely depressed profiles on Twitter. We employed them for demographic (age and gender) inference processes. We developed a multimodal framework, employing statistical techniques for fusing heterogeneous sets of features obtained by processing visual, textual, and user interactions. Conducting an extensive set of experiments, we assessed the predictive power of our multimodal framework while comparing it against state-of-the-art approaches for depressed user identification on Twitter. The empirical evaluation shows that our multimodal framework is superior to them and it improved the average F1-Score by 5 percent. Effectively, visual cues gleaned from content and profile images shared on social media can further augment inferences from textual content for reliable determination of depression indicators and diagnoses. In the future, we plan to apply our approach to various data sources such as longitudinal electronic health record (EHR) systems, and private insurance reimbursement and claims data, to develop a robust “big data” platform for detecting clinical depressive behavior at the community level.

## Supporting information

S1 File The informed consent of this study approved by Wright State University Institution review Board (SC#6258). (PDF)
