# Supplementary material for: Multimodal mental health analysis in social media
Source: PLoS One. 2020 Apr 10;15(4):e0226248. doi: 10.1371/journal.pone.0226248 (PMC7147779; doi:10.1371/journal.pone.0226248)
Supplement: S1 File — (PDF) [file pone.0226248.s001.pdf]

WRIGHT STATE UNIVERSITY

Office of Research and Sponsored Programs
201J University Hall
3640 Col. Glenn Hwy.
Dayton, OH 45435-0001
(937) 775-2425
(937) 775-3781 (FAX)
e-mail: rsp@wright.edu

DATE: July 21, 2016

TO: Amit Sheth, Ph.D., Faculty
Kno.e.sis
Krishnaprasad Thirunarayan, Ph.D., Faculty

FROM: Jodi Blacklidge
Program Facilitator, IRB-WSU

SUBJECT: SC# 6258
'Modeling Social Behavior for Healthcare Utilization and Outcomes in Depression'

The above-listed project does not meet the Federal definition for human subjects research, specifically "a systematic investigation designed to contribute to generalizable knowledge". Therefore, the project does not require approval from the Wright State University Institutional Review Board.

If you have any questions or require additional information, please contact me at 775-3974.

Best wishes for a successful project.
